# Flavokawain B induced cytotoxicity in two breast cancer cell lines, MCF-7 and MDA-MB231 and inhibited the metastatic potential of MDA-MB231 via the regulation of several tyrosine kinases *In vitro*

**DOI:** 10.1186/s12906-016-1046-8

**Published:** 2016-02-27

**Authors:** Nadiah Abu, M. Nadeem Akhtar, Swee Keong Yeap, Kian Lam Lim, Wan Yong Ho, Mohd Puad Abdullah, Chai Ling Ho, Abdul Rahman Omar, Jamil Ismail, Noorjahan Banu Alitheen

**Affiliations:** Bright Sparks Unit, Universiti Malaya, 50603 Kuala Lumpur, Malaysia; Faculty of Biotechnology and Bimolecular Sciences, Universiti Putra Malaysia, UPM Serdang 43400, Selangor Darul Ehsan, Malaysia; Faculty of Industrial Sciences & Technology, Universiti Malaysia Pahang, Lebuhraya Tun Razak 26300, Kuantan, Pahang Malaysia; Institute of Bioscience, Universiti Putra Malaysia, UPM Serdang 43400, Selangor Darul Ehsan, Malaysia; Faculty of Medicine and Health Sciences, Universiti Tunku Abdul Rahman, Lot PT 21144, Jalan Sungai Long, Bandar Sungai Long, Cheras, 43000 Selangor, Malaysia; School of Biomedical Sciences, University of Nottingham Malaysia Campus, Jalan Broga, Semenyih 43500, Selangor, Malaysia

**Keywords:** Breast cancer, Flavokawain, Proliferation, Metastasis, Tyrosine kinase

## Abstract

**Background:**

The kava-kava plant *(Piper methysticum)* is traditionally consumed by the pacific islanders and has been linked to be involved in several biological activities. Flavokawain B is a unique chalcone, which can be found in the roots of the kava-kava plant. In this study, the operational mechanism of the anti-cancer activity of a synthetic Flavokawain B (FKB) on two breast cancer cell lines, MCF-7 and MDA-MB231 was investigated.

**Method:**

Several in vitro assays were attempted such as MTT, flow cytometry of cell cycle analysis, annexin V analysis, and JC-1 analysis to detect apoptosis. Moreover, *in vitro* metastasis assays were also performed such as transwell migration assay, invasion assay, rat aorta ring and HUVEC tube formation. Molecular analysis of related genes and proteins were conducted using real-time PCR and proteome profiler analysis.

**Results:**

Based on our results, apoptosis was induced when both MCF-7 and MDA-MB231 were treated with FKB. A significant G2/M arrest was seen in MDA-MB231 cells. Additionally, FKB also inhibited the *in vitro* migration and invasion in MDA-MB231 cells in a dose dependent manner. Moreover, FKB can be a potential inhibitor in angiogenesis as it suppressed the formation of vessels in HUVEC cells as well as in the *ex-vivo* rat aortic ring assay.

**Conclusion:**

Our findings suggested that FKB also regulated several receptor tyrosine kinases. Overall, FKB is not only a potential candidate to be an anti-cancer agent, but as an anti-metastatic agent as well.

**Electronic supplementary material:**

The online version of this article (doi:10.1186/s12906-016-1046-8) contains supplementary material, which is available to authorized users.

## Background

Cancer is still a worrying disease despite the progressive scientific research conducted. In fact, breast cancer still remains one of the most deadliest cancer among women [1]. Approximately around 1 out of 8 women will develop breast cancer at some point in their lives [1]. According to the cancer statistics reported, around 14 % of breast-cancer related cases ended up being fatal [1]. Additionally, breast cancer has a tendency to become malignant [1]. More than half of the patients diagnosed with breast cancer have already developed metastatic breast cancer [2]. Metastasis is usually the main cause of cancer-related deaths [2, 3]. Therefore, it is extremely crucial to not only treat, but also prevent this disease from becoming malignant. Natural-derived drugs have become a more favourable option in the pharmaceutical industry [4, 5]. In fact, in the development of new anti-cancer drugs, natural products still remain as one of the major players in providing the database [6].

The kava-kava plant extract has been used as traditional remedies to treat various illnesses [7]. Interestingly, there has been a correlation between the consumption of the kava extracts with the incidence of cancer [8]. Some of the most interesting components that can be found from the kava root extracts include the flavokawains [9]. There are three types of flavokawains that can be found and the differences lies in the side chain [10]. Flavokawain B has gained worldwide interest due to its promising anti-cancer activity as reported by several studies [11, 12]. This chalcone was reported to be present in two other types of plant species; the *alpinia* and *piper* family [9, 13]. Flavokawain B was found to possess several fascinating biological activities such as, anti-inflammatory, antinociceptive and anti-cancer activities [13, 14].

The fact that receptor tyrosine kinases (RTK) play an important role in cancer progression was an impactful discovery [15]. There are various RTKs and downstream proteins involved in breast cancer and metastasis such as VEGF, MMP9, GLUT1 and FOXM1. Flavonoids have been reported to regulate some of these RTKs and thus have the potential to become promising anti-cancer agents. Nevertheless, to the best of our knowledge, studies on the effects of flavokawain B on breast cancer cells in vitro and on the RTKs have not yet been reported. Therefore, this study aims to elucidate the anti-metastatic effects of flavokawain B on breast cancer cells in vitro.

## Results

### Flavokawain B inhibited the proliferation of MCF-7 and MDA-MB231 *in vitro*

**Fig. 1:**
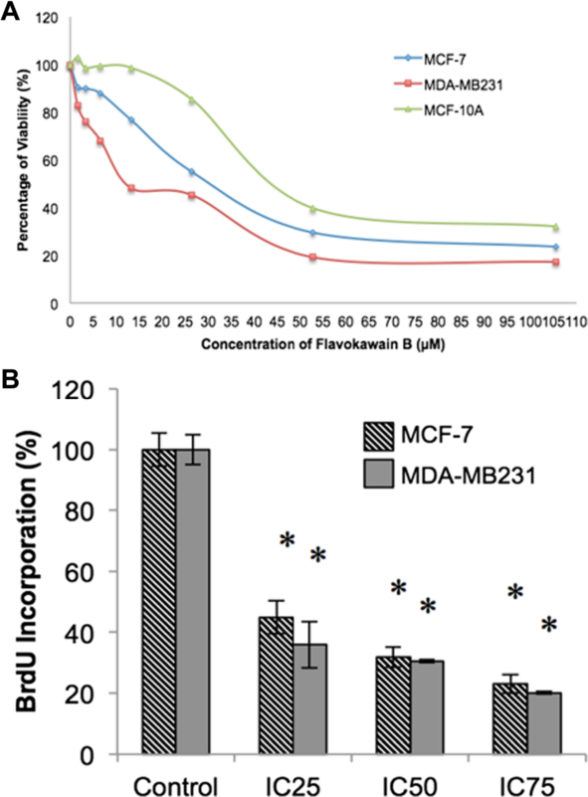
**a** Percentage of viability of three cell lines (MCF-7, MDA-MB231 and MCF-10a) when treated with seven doses of flavokawain B measured through the MTT assay after 72 h. **b** Percentage of BrdU incorporation in both MCF-7 and MDA-MB231 upon treatment with three different concentrations of flavokawain B for 48 h. All data are expressed as mean ± S.E.M with three biological replicates

MTT analysis was performed to analyze the cytotoxic effects of flavokawain B on two breast cancer cell lines, MCF-7 and MDA-MB231, as well as on the non-transformed mammary epithelial cell line, MCF-10A. The cells were treated with 7 different concentrations of flavokawain B for 72 h. As in Fig. 1a, the half-inhibitory concentration (IC_50_) of flavokawain B on MDA-MB231 (12.3 μM) was much lower compared to MCF-7 (33.8 μM). Nonetheless, the IC_50_ of flavokawain B on the positive control cell line, MCF-10A was considerably higher than the two cancer cell lines. The BrdU incorporation assay was also conducted to analyze the anti-proliferative effects of flavokawain B. Fig. 1b depicts that the percentage of BrdU incorporation is negatively correlated with the dose of flavokawain B. Flavokawain B impeded the proliferation of both MCF-7 and MDA-MB231 in a dose-dependent manner.

### Flavokawain B induced G2/M arrest and apoptosis in MDA-MB231 and MCF-7

**Fig. 2:**
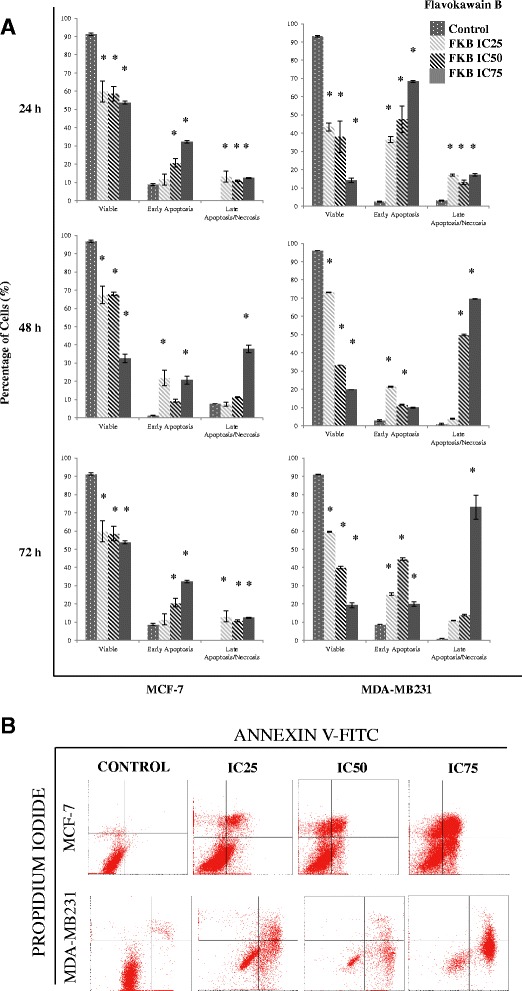
**a** Histogram cell cycle analysis of MCF-7 and MDA-MB231 after 12 h and 24 h of treatment with three different doses of flavokawain B. **b** Representative histogram analysis of the Annexin V-FITC/PI apoptosis assay via FACS Calibur. Both MCF-7 and MDA-MB231 were treated with three different concentrations of flavokawain B for 48 h. (Quantitative analyses of cell cycle and annexin V assays are presented in Additional files 1 and 2: Figures S1 and S2 respectively. All data are expressed as mean ± S.E.M with three biological replicates)

To investigate the effects of flavokawain B on the cell cycle progression in MDA-MB231 and MCF-7, the FACS cell cycle analysis was performed. Three different concentrations of flavokawain B were used as treatment and the cells were analysed at 12 h and 24 h post-treatment. Based on Fig. 2 the changes in the G2/M phase in MDA-MB231 cells were significant as the dose increases. After 24 h, the percentage of cells in the sub G0/G1 phase also increased as the dose escalates. In MCF-7 cells however, there was no significant G2/M arrest seen at both time points, as depicted in Fig. 2a (Additional file 1: Figure S1).

One paramater of apoptosis was examined by detecting the externalization of phosphatidylserine. The cells were stained with Annexin V-FITC and Propidium Iodide before being subjected to flow cytometry. According to Fig. 2b (Additional file 2: Figure S2), in both MCF-7 and MDA-MB231 a pattern can be seen where there is a shift in the percentage of cells from viable to early apoptosis and to late apoptosis/necrosis as the dose escalates.

### Flavokawain B inhibited migration and invasion in vitro and suppressed the formation of tube-like vessels in vitro and ex vivo

**Fig. 3:**
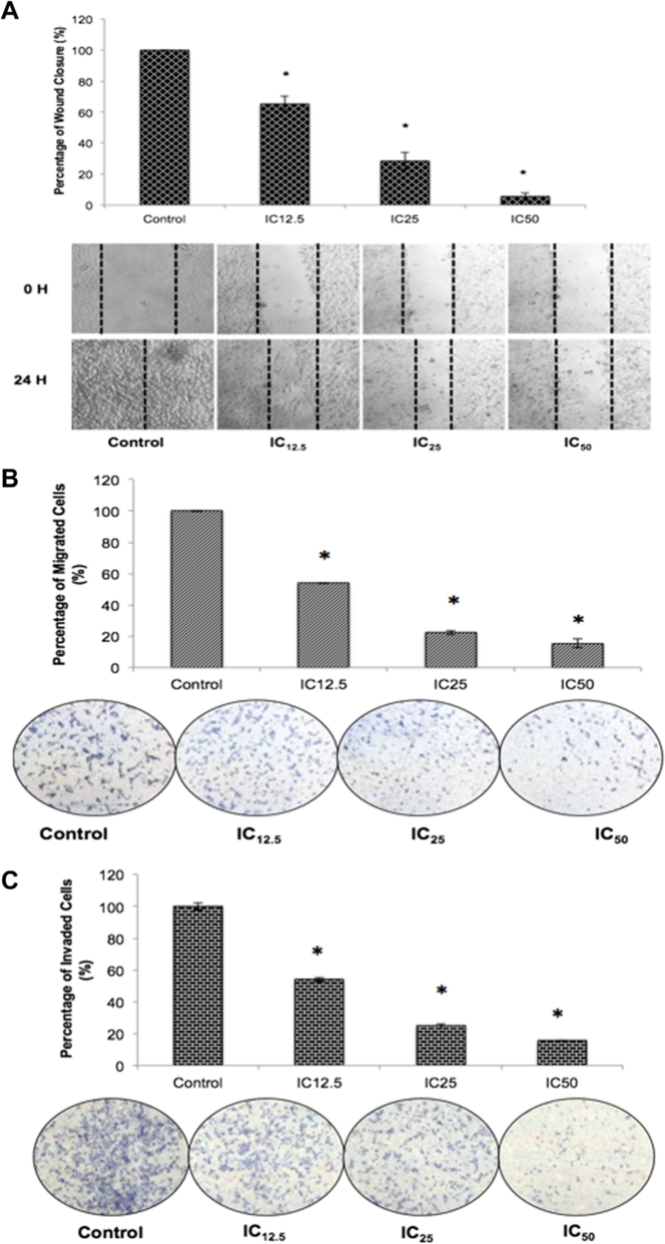
**a** Percentage of wound closure in MDA-MB231 cells after treatments with three different concentrations of flavokawain B. Representative images of the wound in MDA-MB231 at 0 hourand 24 h upon treatment with flavokawain B. **b** In vitro matrigel-transwell invasion assay was performed on MDA-MB231 treated cells after being serum-starved for 24 h. Cells were allowed to invade an extracellular matrix (matrigel) concurrently being treated with three different doses of flavokawain B. **c** In vitro transwell migration assay was conducted in MDA-MB231 cells when treated with three different concentrations of flavokawain B for 24 h. Cells were allowed to migrate through an 8 μM pore with FBS as a chemo-attractant. Magnification: 20X. Data are expressed as mean ± S.E.M. (*p* < 0.05)

For the in vitro motility assessment, the scratch (wound healing) assay was used in MDA-MB231. The percentage of wound closure was compared between all treatments of group with the control as showed in Fig. 3a. In the control group of the MDA-MB231 cells, the area of the wound was completely covered with migrated cells after 24 h (100 ± 0.1 %). In the treated groups, the percentage of wound closure is negatively correlated to the dose of flavokawain B given. For the IC_12.5,_ IC_50_ and IC_75_treated groups, the wound closure is approximately 65 ± 4.9, 28.6 ± 5.6 and 5.4 ± 2.7 % respectively.

The transwell assay was also performed to investigate the effects of flavokawain B on the motility and invasiveness of MDA-MB231. The percentage of migrated cells was inversely proportional to the dose of flavokawain B according to Fig. 3b. In MDA-MB231 treated cells, the higher the dose of flavokawain B; the lower the percentage of migrated cells through the transwell. For the IC_50_ treated group, the percentage of migrated cells in MDA-MB231 was 15.4 %. The matrigel-transwell assay was conducted using MDA-MB231 cells only since it is highly invasive as compared to MCF-7. Similar to the in vitro migration assay, as well as the wound-healing assay, the percentage of invaded cells is negatively correlated to the dose of flavokawain B. In the flavokawain B-treated groups, IC_12.5_, IC_25_ and IC_75_ the percentage of invaded cells were 54.1, 24.7 and 15.5 % correspondingly as in Fig. 3c.

**Fig. 4:**
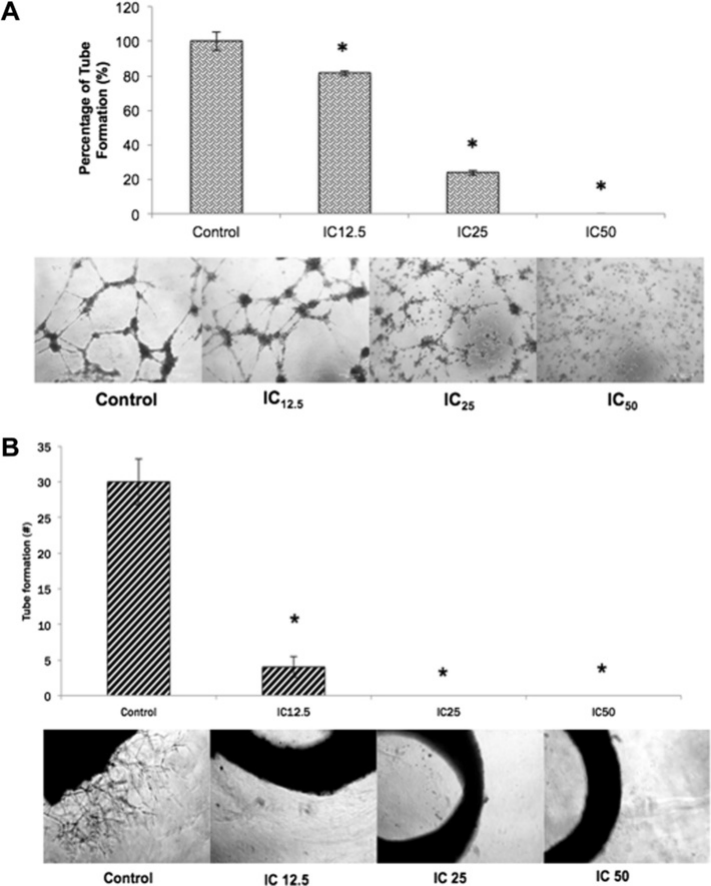
**a** Tube formation analysis of HUVEC cells when treated with three different doses of flavokawain B for 18 h. Experiment was done in triplicates and all data are expressed as mean ± S.E.M. Magnification: 20X. **b** Sprouting of vessels from the ex-vivo rat aortic ring assay following the treatment of three different doses of flavokawain B. The aortas were excised from Sprague-drawley rats and the aorta was incubated for 7 days. Experiment was done in triplicates and all data are expressed as mean ± S.E.M. Magnification: 10X

For the in vitro angiogenesis assessment, the HUVEC tube formation assay was conducted, followed by treatment with three different concentrations of flavokawain B. In the IC_12.5_ treated group, there was no significant inhibition in the formation of tube-like vessels as represented in Fig. 4a. The percentage of tube formation in IC_25_-treated group was significantly suppressed with 23.6 % tube formation. There was a total inhibition in the formation of tube in the IC_50_-treated group. The ex-vivo rat aortic ring assay was also conducted to further understand the anti-angiogenic potential of flavokawain B as shown in Fig. 4b. This also suggests that flavokawain B regulates angiogenesis in a dose-dependent manner.

### Flavokawain B regulated several metastasis-related proteins and genes, and tyrosine kinases in MDA-MB231

**Fig. 5:**
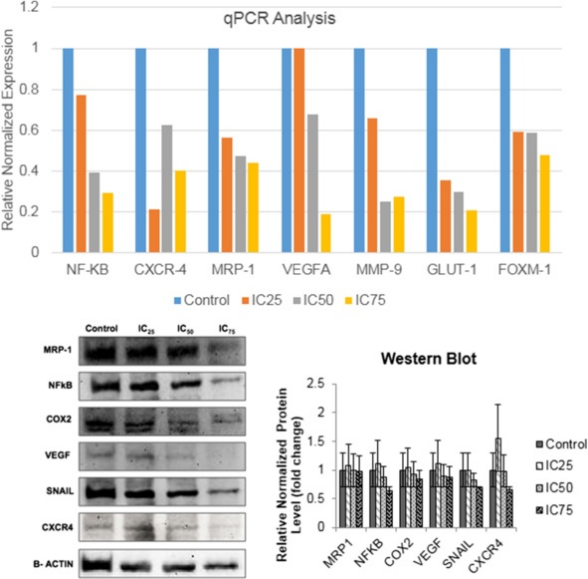
Relative mRNA expression level of metastasis-related genes; MMP9, VEGF, GLUT1, NF-KB, CXCR4, MRP1 and FOXM1 in MDA-MB231 cells 48 h post-treatment with three concentrations flavokawain B for 18 h. Experiment was done in triplicates and all data are expressed as mean ± S.E.M. Protein expression of several metastasis-related proteins; MRP-1, NF-kb, COX-2, VEGF, SNAIL and CXCR4 in MDA-MB231 cells 48 h post-treatment with three doses of flavokawain B

To measure the relative mRNA level of metastasis-related genes, quantitative real-time analysis was performed as displayed in Fig. 5. Upon induction with three doses of flavokawain B, the mRNA expression of all genes in MDA-MB231 treated cells; MMP9, VEGF, GLUT1 and FOXM1 were down regulated. The significance of these findings were dose-dependent; the lower the dose of flavokawain B given, the less significant the expression of mRNA. The expressions of the mRNA level were considered significant if it were more than 2-fold. In the western blot analysis on the other hand, the protein expression of inflammation-related proteins such as NF-KB and COX-2 were decreased in a dose-dependent manner. Likewise, the same pattern can be seen in other metastasis-related proteins such as VEGF, SNAIL and CXCR4 according to Fig. 5.

**Fig. 6:**
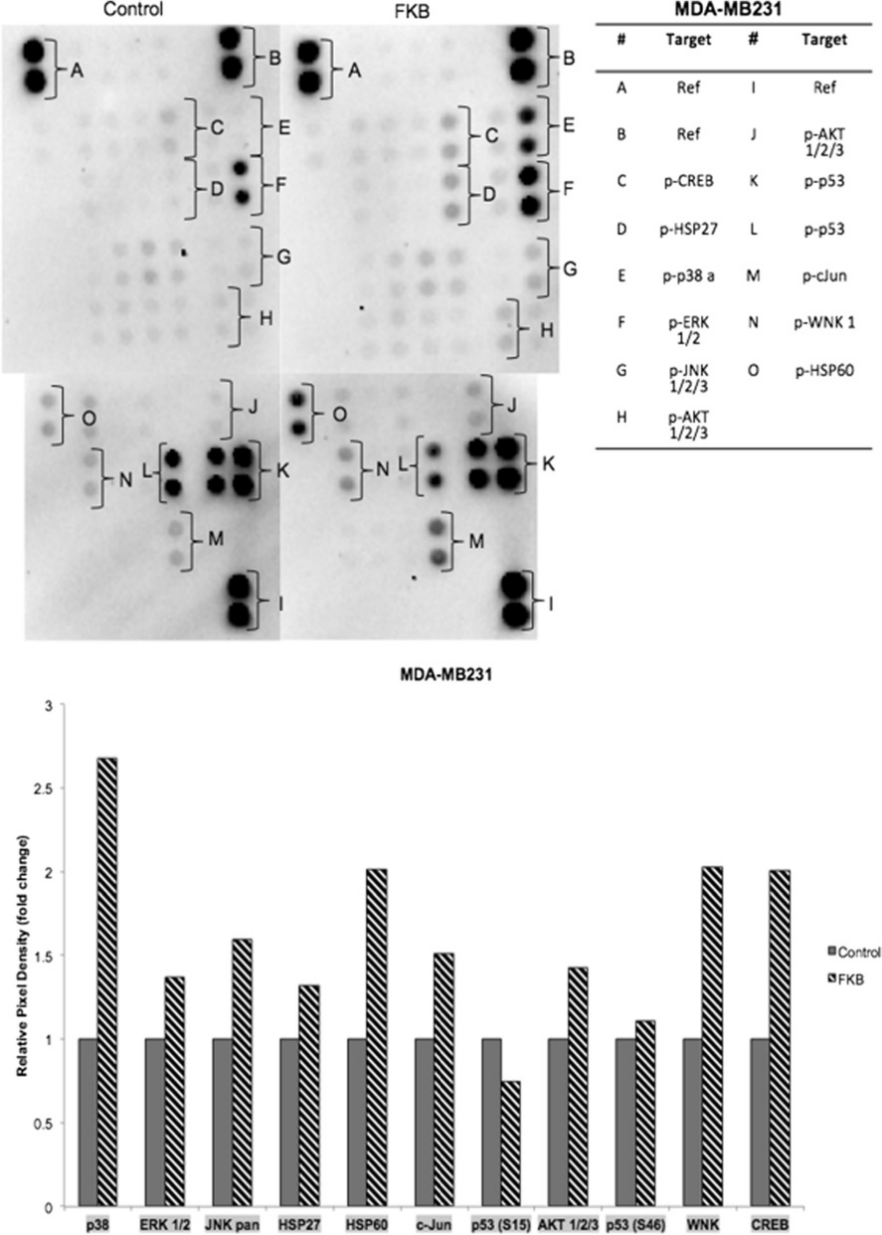
Proteome Profiler ^TM^ using Phospo-RTK using MDA-MB231 cell lines treated with flavokawain B for 48 h. Experiment was done in triplicates and all data are expressed as mean ± S.E.M

Based on the proteome profiler analysis, flavokawain B regulated several important tyrosine kinases in MDA-MB231. In Fig. 6, in MDA-MB231, flavokawain B upregulated the phosphorylation of p-p38 alpha, p-CREB, p-HSP27, p-JNK, p-AKT, p-ERK, p-HSP60, p-WNK1, p-c-Jun and p-p53.

## Discussion

From our findings, it can be suggested that FKB induces apoptosis and inhibits proliferation in both MCF-7 and MDA-MB231 cells in vitro as well as halting some of the steps in the metastatic cascade in MDA-MB231 cells in vitro*.* FKB possess anti-proliferative effects and is selective in its actions as evidenced by the MTT and BrdU assays. It has been documented in some studies that FKB induced G2/M arrest in several cancer cell lines [12–14]. It is observed that FKB induced G2/M arrest in MDA-MB231 cells in the early stages of treatment. Metastasis accounts for the major cause of cancer-related mortalities especially in breast cancer patients [16]. Therefore it is essential to not only inhibit the growth of cancer cells, but hamper the metastatic process as well. The ability of cancer cells to migrate and invade extracellular tissues contributes greatly to the likelihood of forming secondary tumors. Both migration and invasion are crucial steps in metastasis and FKB was shown to impede both of these processes in vitro dose-dependently. Besides that, angiogenesis, the formation of blood vessels, is also an essential step in metastasis because the formation of secondary tumors is highly dependent on the adequacy of blood and nutrient supply [17]. FKB was not only able to impede the motility and invasiveness of MDA-MB231 in vitro, but also thwart the formation of vessel-like structures in vitro and ex vivo.To further understand the role of FKB in suppressing metastasis, the expression of 4 metastasis-related genes were measured in FKB-treated cells. MMP9 is a matrix metalloproteinase protein that is actively involved in metastasis, especially relating to angiogenesis [18, 19]. In breast cancer particularly, MMP9 has been found to be expressed in tumors expanding to secondary sites [20]. The down regulation of MMP9 could decrease the metastatic ability of cancer cells. Apart from that, cancer cells have a high demand of energy and increased metabolism; therefore the need of substantial energy is required [21]. The Warburg effect is a popular doctrine regarding the active level of aerobic glycolysis in cancer cells [22]. GLUT1 is a glucose transporter that plays an important role in the uptake of glucose by glycolysis in tumor and is often associated in malignant phenotypes of cancer [22]. In FKB-treated cells, the expression of GLUT1 at the mRNA level is decreased in both cell lines. One of the most infamous biomarkers used to detect metastasis is VEGF. Most often, VEGF is overexpressed in tumors as it promotes angiogenesis as well as accelerating metastasis [23]. Moreover, FOXM1 is a transcription factor involving in cell cycle progression [24]. Nevertheless, it has been noted that FOXM1 also plays a role in the development of cancer on a wider scale [25]. FOXM1 has been found to promote metastasis in association with MMP9 as well as VEGF [24]. In FKB-treated cells, it can be seen that FOXM1 was down regulated in all treated groups in a dose-dependent manner.

Cancer progression as well as the metastatic process itself is often associated with inflammation [26, 27]. In fact, it has been put forward that inflammation actually accelerates the metastasis process altogether [27]. NF-KB is a well-known protein to be involved in inflammation and cancer. In fact, through the inhibition of NF-KB in a breast cancer model, the EMT process, a subset in the whole metastatic cascade, is decreased [28]. From our results, it can be suggested that FKB decreases the protein expression of NF-KB depending on the dose given. This overall implies that FKB may inhibit the inflammatory process in cancer cells, and eventually metastasis. Additionally, FKB was also found to impede the expression of COX-2, another inflammation-related protein. In breast cancer specifically, the suppression of COX-2 has been a targeted therapeutic approach in treating this disease [29, 30]. As seen in FKB-treated cells, similar to the expression of NF-KB, there was a decrement in the level of expression in COX-2 as well. There are several key regulators in the EMT process including E-cadherin, Snail, Slug and Twist [31]. In fact, the pro-inflammatory protein, NF-KB is also commonly associated with the activation of EMT; NF-KB is involved in EMT by regulating and stabilizing the protein Snail. It has been studied by the depletion of Snail; the manifestation of metastasis is reduced both in vitro and in vivo [31]. Moreover, breast cancer is regularly known to metastasize to the bones, lymph nodes and lungs. One of the major important players in this process is the CXCR4 [32]. CXC-chemokine receptor 4 (CXCR4) belongs to the G-protein coupled receptor group and is regularly involved in breast cancer metastasis [32].

Tyrosine kinases play major roles in the apoptosis and metastasis pathways. Based on the proteome profiler results in MDA-MB231, the phosphorylation of p38alpha increased in the FKB-treated group. P38 mitogen-activated protein kinase is regularly involved in cell proliferation, survival and differentiation [33]. Additionally the phosphorylation of p38 MAPK is known to lead to the activation of several other important molecules including heat shock protein 27 [34]. HSP27 was significantly up regulated in the FKB-treated cells. This heat shock protein is activated by several other factors including TNF-alpha, interleukins and growth factors [34]. HSP27 has dual roles in cancer progression, it has been reported that HSP27 inhibits the in vitro proliferation of several cancer cells but nonetheless, it also affects the metastatic phenotype [35]. P38 is also involved in the regulation of other cell cycle associated proteins such as p53 and ATF2 [36, 37]. Additionally, it can be seen that there is a difference in the level of expression of p-CREB. This protein is the downstream product of the p38, ERK1/2 and AKT pathways. CREB is responsible for the transcription of several survival-related proteins. The p38 MAPK pathway is also commonly intertwined with the JNK and ERK pathway. JNK is also a major MAPK family that is activated upon stress. Downstream of this pathway is c-Jun, and it is known to inhibit proliferation and induce apoptosis in cancer cells [38, 39]. Both levels of phosphorylated JNK and c-Jun were increased upon treatment with FKB. Additionally, besides p38 and JNK, the ERK MAPK pathway was also activated. This pathway is known to have dual roles back and forth between being anti-apoptotic and pro-apoptotic [40]. Persistent activation of the ERK ½ pathway is also known to have significant effects on the cell cycle machinery and MMP9, as it is evident in FKB-treated cells [41]. This eventually led to the deregulation of NF-KB and COX2 [42]. Moreover, the level of HSP60 was increased in MDA-MB231 upon treatment with FKB. HSP60 is a heat shock protein involved in ambiguous pro-apoptotic and pro-survival functions in cancer cells [43].

## Materials and methods

### Synthesis of flavokawain B

Flavokawain B used in all experiments was synthesized by the reaction of 4′,6′-dimethoxy-2′-hydroxyacetophenon and benzaldehyde. Acetophenone and aldehyde were mixed together with 40 % solution of KOH. The detailed synthesis, purification and characterization of flavokawain B has already been explained in our previous publication [34]. The purity of FKB was 99.9 %. Below is the NMR data obtained after synthesizing FKB:

- (Flavokawain B) *(E)-1-(2′-Hydroxy-4′,6′-dimethoxyphenyl)-3-phenylprop-2-en-1-one*
- (*E*)-1-(2′-Hydroxy-4′,6′-dimethoxyphenyl)-3-phenylprop-2-en-1-one: yellow needles crystals: yeild 82.4 %, m.p. 102–104 °C, EI-MS m/z 284.23, (molecular formula C_17_H_16_O_4_). ^1^HNMR (CDCl_3_, 500 MHz). δ 7.92 (d,1H, *J* =14.5 Hz,Hβ), 7.80 (d,1H, *J* = 14.5 Hz, Hα), 7.62 (br,d, 2H, H-2,6), 7.46 (m, 3H,H-3, 4, 5), 6.12 (br,s, 1H, H-3′), 5.98 (br,s, 1H, H-5′), 3.93 (s, 3H, OMe, C-6′), 3.85 (s, 3H, OMe, C-4′).

### Ethical approval

According to the Universiti Putra Malaysia’s Institutional Animal Care and Use Committee (IACUC), the usage of cell lines obtained from a commercial source does not require an ethical approval from the committee. For the ex vivo assays, the experiments were done in accordance to the Institutional Animal Care and Use Committee guidelines. The ethical approval for the ex vivo study was sought and obtained from the Universiti Putra Malaysia’ Institutional Animal Care and Use Committee (IACUC).

### Cell culture

The cell lines MCF-7, MDA-MB231 and MCF-10A were obtained from the ATCC collection (ATCC, Rockville, MD, USA). MCF-7 was maintained in RPMI supplemented with 10 % fetal bovine serum (FBS), while MDA-MB231 was maintained in DMEM, supplemented with 10 % FBS. MCF-10A on the other hand, was maintained in DMEM-F12 media supplemented with hydrocortisone (0.5 μg/ml), insulin (10 μg/ml), hEGF (20 ng/ml) and 10 % (v/v) FBS. All the cells were kept in a 37 °C incubator with 5 % CO2.

### MTT assay

This assay was performed as outlined by Mosmann (1983) [45] with minimal modifications. The cells were seeded in a 96-well plate at a concentration of 0.8x10^5^ cells/ml. Flavokawain B was added to the wells at the desired concentrations one day after the seeding. After the designated incubation time, MTT solution (5 mg/ml) was added at a volume of 20 μl in each well and the cells were incubated for an additional three hours. Then, the solution was removed, and 100 μl of DMSO was added to solubilize the crystals. The plates were then read using the μquant plate reader at the wavelength of 570 nm (Bio-tek instruments, USA). The following method of calculation was used to determine the percentage of viable cells:

$$ \mathrm{Percentage}\ \mathrm{of}\ \mathrm{Cell}\ \mathrm{Viability} = \left[{\mathrm{A}}_{570}\mathrm{Control}\ /{\mathrm{A}}_{570}\mathrm{Sample}\right]\ \mathrm{x}\ 100\ \% $$

### Cell treatment

Based on the MTT assay, subsequent assays were performed according to the results obtained. FKB was dissolved in DMSO and the percentage of administration to the cells was less than 0.1 %. Three different concentrations of flavokawain B were used in the treatment of MCF-7 and MDA-MB231 as outlined in Table 1. The control sample was treated with the vehicle (DMSO) at 0.1 %.

**Table 1:** The doses of flavokawain B used in the treatment of MCF-and MDA-MB231 in all of the assays

Cell line		
Flavokawain B (μM)	MCF-7	MDA-MB231
IC_25_	10.5	6
IC_50_	31.1	12.3
IC_75_	40.5	24.6

### BrdU incorporation assay

The BrdU incorporation was measured using the BrdU Cell Proliferation Kit (Merck, Germany). The cells were seeded in a 96 well plate at a concentration of 0.8x10^5^ cells/ml and left overnight. The next day, the cells were treated with three different concentrations of flavokawain B. 24 h before fixing the cells, 20 μl of BrdU was added to each well. After the respective incubation hours, the cells were fixed and denatured using a fixing solution. The plates were then stored at 4 °C. Then, the plates were washed twice before adding 100 μl of the detector antibody into each well for 1 h. Next, 100 μl of Goat anti-mouse Ig G-HRP conjugated was added for 30 min. Afterwards, the plates were incubated with 100 μl of the TMB substrate for roughly 30 min. Finally, 100 μl of stop solution was added and the absorbance was measured at 450 nm, using the μquant plate reader (Bio-tek Instruments, USA).

### Cell cycle analysis

The cells were seeded in 6 well plates at a density of 2.4x10^5^ cells/ml. The following day, the cells were treated with three different concentrations of flavokawain B. After 12 and 24 h of treatment, the cells were detached using trypsin, washed with PBS and collected. The resulting pellet was fixed in 80 % ethanol and stored at −20 °C. After a week, the fixed cells were washed with PBS and treated with RNAse and Triton-X 100, and were then stained with propidium iodide (PI) for 15 min (Sigma, St Louis, MO, USA). Afterwards, the cells were subjected to flow cytometric analysis using the FACS Calibur flow cytometer (Becton Dickinson, USA).

### Annexin V/FITC assay

The Annexin V assay was carried out using the Annexin V Kit (BD Pharmingen, USA). Cells were seeded in 6 well plates (2.4x10^5^ cells/ml) and were treated with the desired concentrations of flavokawain B. After 24, 48 and 72 h of treatment, the treated cells were collected and harvested according to the desired time points. The resulting pellets were immediately resuspended in the provided binding buffer and subsequently stained with 5 μ lof Annexin V-FITC and 5 μl of PI. The subsequent mixture was left to incubate at room temperature for 15 min. Afterwards, the cells were analyzed using the FACS system (Becton Dickinson, Franklin Lakes, NJ, USA).

### Wound healing assay

The cells were seeded in 6 well plates to total confluence for 24 h. On the day of the treatment, a wound was introduced in the middle of the wells using a sterile yellow tip pipette. Afterwards, the media was replaced with fresh media along with the addition of the designated compounds. The migration of cells was photographed at 10x magnification for every 3 h up until 24 h. The rate of migration was calculated based on the following formula.

$$ \%\ \mathrm{Percentage}\ \mathrm{of}\ \mathrm{Wound}\ \mathrm{closure} = \left(\mathrm{Length}\ \mathrm{of}\ \mathrm{wound}\ \mathrm{at}\ \left(0\ \mathrm{h}-\left(\mathrm{n}\right)\ \mathrm{h}\right)/\mathrm{Length}\ \mathrm{of}\ \mathrm{wound}\ \mathrm{at}\ 0\ \mathrm{h}\right)\ *100 $$

### Transwell migration/Invasion assay

The transwell migration/invasion assay was performed using an 8 μM pore cell culture insert (BD, USA). Matrigel (BD, USA) was diluted at a ratio of 1:3 with serum-free media and was applied to the top chamber of the cell culture inserts for the invasion assay, whereas in the migration assay, the inserts were not coated. The matrigel-coated inserts were left for 2 h to solidify before being used. Next, 2x10^5^ cells/ml were seeded in the upper chamber of the inserts after being serum-starved for 24 h. The lower chamber was filled with media containing 10 % of FBS. The cells were incubated in a humidified chamber at 37 °C for 24 h. Afterwards, the non-migrating/invading cells were removed using a cotton swab. The migrated/invaded cells were fixed in methanol for 30 min and were stained with crystal violet for 30 min before being photographed.

### HUVEC tube formation assay

This assay was performed as described by Arnaoutova et al. [46] with slight modifications. Prior to the assay, 50 μl of Matrigel (BD, USA) was seeded in a 96 well plate for 2 h. HUVEC cells at a density of 1 x10^5^ cells/ml were seeded in the pre-coated 96 well plates alongside the treatments. The cells were incubated in a 37 °C-humidified chamber for 18 h before being photographed under an inverted light microscope (Nikon, Japan) at 40x magnification.

### Rat aortic ring assay

For this experiment, 6-weeks old, male Sprague Drawley rats were used. Freshly removed aortas were rinsed in ice-cold PBS supplemented with penicillin-streptomycin before being cut to 1 mm-1.5 mm segments. The segments were placed on top of a matrigel-coated 48 well plate. Another aliquot of 150 μl of ice-cold matrigel was placed on top of the segments to sandwich it in between two layers of matrigel, and was allowed to solidify for 2 h at 37 °C. The segments were incubated in ECM media (Sciencell, USA) for 24 h before being replaced with fresh ECM media containing three different concentrations of Flavokawain B. The aortic rings were photographed on day 7 using an inverted light microscope (Nikon, Japan). The Animal Care and Use Committee, Universiti Putra Malaysia (UPM/FPV/PS/3.2.1.551/AUP-R168) approved this study according to the guidelines from the committee.

### Quantitative real-time PCR

After the treatment with flavokawain B, total RNA was isolated using the RNaeasy Kit according to the manufacturer’s protocol (Qiagen, Germany). The purity and concentration of the isolated RNA were measured using a Nano-spectrophotometer (Beckman Coulter, USA). Next, 1 μg of the RNA was converted to cDNA using the QuantiTect Reverse Transcription Kit according to the manufacturer’s protocol (Qiagen, Hilden, Germany). Afterwards, real-time PCR was carried out using the SYBRSelect Master Mix (Invitrogen, USA) on the iCycler IQ5 (Bio-Rad, Hercules, USA). The program for the PCR reaction was initiated at 95 °C for 10 min. This was followed by 40 cycles of denaturation at 95 °C for 10 s and annealing/extension step at 60 °C. Table 2 illustrates the gene, accession number and sequence of the primers used. The analysis of the qPCR results were performed based on the efficiency of the primers using the GeNorm analysis and two housekeeping genes (GAPDH and Beta-actin) to normalize. The difference in the fold change was analysed between the normalized untreated (control) and FKB-treated samples.

**Table 2:** Sequence, accession number, length of amplicon and the name of the gene of the primers used in the qPCR analysis

Accession number	Gene	Amplicon length	Sequence
NM_001101.3	ACTB	184	F: 5′-AGAGCTACGAGCTGCCTGAC-3′
			R: 5′-AGCACTGTGTTGGCGTACAG-3′
NM_002046.4	GAPDH	206	F: 5- GGATTTGGTCGTATTGGGC-3
			R: 5- TGGAAGATGGTGATGGGATT-3
NM_001287044.1	VEGF	195	F: 5-GCTGTGGACTTGAGTTGGG-3
			R: 5-GCTGGGTTTGTCGGTGTT-3
NM_001243089.1	FOXM1	175	F: 5-ATACGTGGATTGAGGACCACT-3
			R: 5-TCCAATGTCAAGTAGCGGTTG −3
NM_006516.2	GLUT1	168	F: 5- ACAACACTGGAGTCATCAATGC −3
			R: 5-CCACAGAGAAGGAGCCAATCA-3
NM_004994.2	MMP9	266	F: 5-GAGTGGCAGGGGGAAGATGC-3
			R: 5-CCTCAGGGCACTGCAGGATG-3
NM_019898	MRP1	117	F: 5-GTCGGGGCATATTCCTGGC-3
			R: 5-CTGAAGACTGAACTCCCTTCCT-3
NM_003998	NF-KB	104	F: 5-AACAGAGAGGATTTCGTTTCCG-3
			R: 5-TTTGACCTGAGGGTAAGACTTCT-3
NM_003467	CXCR4	96	F: 5-ACGCCACCAACAGTCAGAG-3
			R: 5-AGTCGGGAATAGTCAGCAGGA-3

### Western blot

Total protein lysates were obtained by lysing the cells with ice –cold RIPA buffer supplemented with phosphatase inhibitor cocktail (Roche, Canada). The quantification of protein was measured using the Bradford assay (Sigma, USA). Next, 100 μg of each sample proteins were subjected to a 10 % SDS-Page before the proteins were transferred onto a PVDF membrane (Roche, Canada) using the Pierce Fast Semi-Dry Blotter (Pierce, USA). Afterwards, the membranes were blocked in 0.5 % skimmed milk overnight. The following day, the membranes were washed in TBST for 10 min to a total of 3 washes and were incubated with the antibodies, anti-SNAIL (ab82846, Abcam, USA), anti-CXCR4 (ab2074, Abcam, USA), anti-VEGF (ab1316, Abcam, USA), anti-COX2 (ab15191, Abcam, USA), anti-NFKB (AHP 1342, Abdserotec, USA), anti-MRP1(ab32574, Abcam, USA). Afterwards, the membranes were incubated in the appropriate secondary antibodies conjugated with HRP. The western blots were then developed under chemiluminescence condition (SuperSignal West Pico, Pierce, USA) using the ChemiDocXRS machine (Bio-rad, USA). The bands were then analyzed using the Quantity One 1D Analysis software (Bio-rad, USA).

### Proteome profiler array ^TM^

The proteome profiler antibody array was employed to determine the effects of flavokawain B on the phosphorylation of several tyrosine kinases. This assay was done according to the manufacturer’s protocol. The cell lysates were incubated with the designated membranes overnight at 4 °C. The following day, the membranes were washed three times and were then incubated with the freshly prepared antibody cocktail for 2 h. Afterwards, the membranes were washed for three times again, before being incubated with the streptavidin-horseradish-peroxidase for 30 min at room temperature. Then, the membranes were developed under chemiluminescence conditions using the ChemiDOC XRS (Bio-rad, USA).

### Statistical analysis

All experiments were done in triplicates and the average values were obtained. The statistical analysis was performed using the GraphPad Prism (version 4.0). For the experimental analysis the one-way ANOVA was performed followed by Tukey’s *post hoc* test. The significance was set at *p* < 0.05. The comparison of statistical significance was done between the control (treated) and flavokawain B treated groups.

## Conclusion

In conclusion, FKB remains a promising anti-cancer agent in treating breast cancer especially metastatic breast cancer. It is in our future interest to do further in-depth study to investigate the effects of FKB on p38 pathway, NRF-2 pathwa, thioredoxin pathway and other cell stress-related pathways. Further studies are required to truly understand the functional machinery of FKB.

## Additional files

Additional file 1: Figure S1. Bar chart analysis of the cell cycle assay in three independent replicates of both MCF-7 and MDA-MB231. All data are expressed as mean ± S.E.M with three biological replicates. (TIF 103 kb) Additional file 2: Figure S2. Bar chart analysis of the Annexin V assay in three independent replicates of both MCF-7 and MDA-MB231 after 24, 48 and 72 h of tretmanet with FKB. All data are expressed as mean ± S.E.M with three biological replicates. (TIF 94 kb)

## Abbreviations

AKT: RAC-alpha serine/threonine-protein kinase
ATF-2: activating transcription factor 2
COX-2: prostaglandin-endoperoxide synthase 2
CREB: cAMP response element-binding protein
CXCR4: C-X-C chemokine receptor type 4
ERK1/2: extracellular-signal-regulated kinases
FKB: flavokawain B
GLUT-1: glucose transporter 1
HSP-27: heat shock protein 27
HSP60: heat shock protein 60.
JNK: c-Jun N-terminal kinases
MAPK: mitogen-activated protein kinases
MMP-9: matrix metallopeptidase 9
MRP-1: multidrug resistance-associated protein 1
NFKB: nuclear factor kappa-light-chain-enhancer of activated B cells
SNAIL: zinc finger protein SNAI1
VEGF: vascular endothelial growth factor
