# Supplementary figures and images for: Flavokawain B induced cytotoxicity in two breast cancer cell lines, MCF-7 and MDA-MB231 and inhibited the metastatic potential of MDA-MB231 via the regulation of several tyrosine kinases In vitro
Source: BMC Complement Altern Med. 2016 Feb 27;16:86. doi: 10.1186/s12906-016-1046-8 (PMC4769841; doi:10.1186/s12906-016-1046-8)

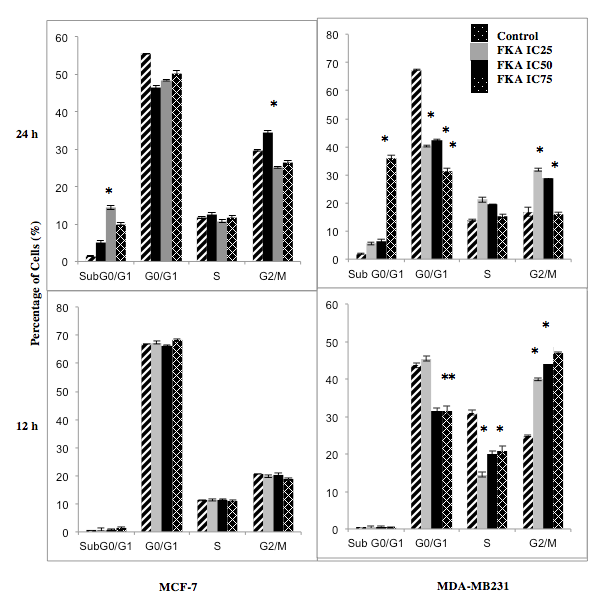

Supplement: Additional file 1: Figure S1. — Bar chart analysis of the cell cycle assay in three independent replicates of both MCF-7 and MDA-MB231. All data are expressed as mean ± S.E.M with three biological replicates. (TIF 103 kb) [file 12906_2016_1046_MOESM1_ESM.tif]

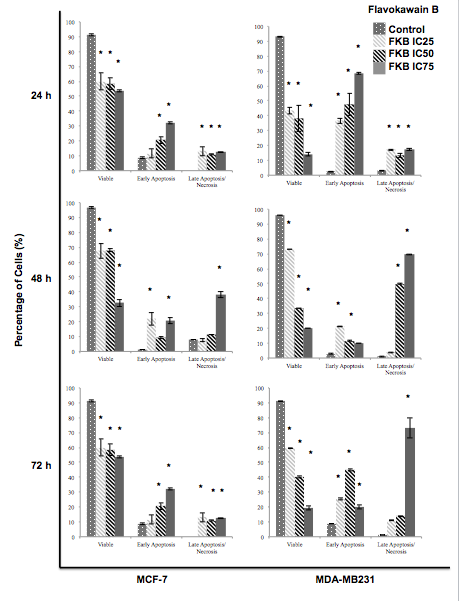

Supplement: Additional file 2: Figure S2. — Bar chart analysis of the Annexin V assay in three independent replicates of both MCF-7 and MDA-MB231 after 24, 48 and 72 h of tretmanet with FKB. All data are expressed as mean ± S.E.M with three biological replicates. (TIF 94 kb) [file 12906_2016_1046_MOESM2_ESM.tif]
